# Quantitative Proteomic and Transcriptomic Analyses of Metabolic Regulation of Adult Reproductive Diapause in *Drosophila suzukii* (Diptera: Drosophilidae) Females

**DOI:** 10.3389/fphys.2019.00344

**Published:** 2019-04-04

**Authors:** Yifan Zhai, Xiaolin Dong, Huanhuan Gao, Hao Chen, Puyun Yang, Ping Li, Zhenjuan Yin, Li Zheng, Yi Yu

**Affiliations:** ^1^Institute of Plant Protection, Shandong Academy of Agricultural Sciences, Jinan, China; ^2^College of Agriculture, Yangtze University, Jingzhou, China; ^3^College of Life Sciences, Shandong Normal University, Jinan, China; ^4^Shandong Academy of Group, Jinan, China; ^5^National Agro-technical Extension and Service Center, Beijing, China

**Keywords:** *Drosophila suzukii*, quantitative proteomics, RNA-Seq transcriptomics, reproductive diapause, qRT-PCR

## Abstract

Diapause is a form of dormancy used by many insects to survive adverse environmental conditions, which can occur in specific developmental stages in different species. *Drosophila suzukii* is a serious economic pest and we determined the conditions for adult reproductive diapause by the females in our previous studies. In this study, we combined RNA-Seq transcriptomic and quantitative proteomic analyses to identify adult reproductive diapause-related genes and proteins. According to the transcriptomic analysis, among 242 annotated differentially expressed genes in non-diapause and diapause females, 129 and 113 genes were up- and down-regulated, respectively. In addition, among the 2,375 proteins quantified, 39 and 23 proteins were up- and down-regulated, respectively. The gene expression patterns in diapause- and non-diapause were confirmed by qRT-PCR or western blot analysis. The overall analysis of robustly regulated genes at the protein and mRNA levels found four genes that overlapped in the up-regulated group and six genes in the down-regulated group, and thus these proteins/genes may regulate adult reproductive diapause. These differentially expressed proteins/genes act in the citrate cycle, insulin signaling pathway, PI3K-Akt signaling pathway, and amino acid biosynthesis pathways. These results provide the basis for further studies of the molecular regulation of reproductive diapause in this species.

## Introduction

Diapause is a form of dormancy characterized by a positive response to changing environmental conditions, which comprises complex physiological and biochemical processes involving many interacting regulatory mechanisms, and it can help many insects and other animals to survive crises (Denlinger, 2002, 2008). In addition, diapause typically occurs in a specific developmental stage in different species, such as the embryo, larvae/nymph, pupa, or adult stages (Jiang et al., 2010; Liu et al., 2010; Kobayashi et al., 2014; Zhai et al., 2016). Adult reproductive diapause often occurs because many insect species overwinter as adults, where the processes of oogenesis and vitellogenesis usually stop (Baker and Russell, 2009).

Spotted wing drosophila (SWD), *Drosophila suzukii* (Diptera: Drosophilidae), is native to Asia. Its western invasion started in 2008 when synchronous outbreaks occurred in North America and Europe (Rota-Stabelli et al., 2013; Cini et al., 2014). SWD has a large host range, and more than 80 plant species serve as the primary hosts. Soft berries and stone fruits (e.g., strawberry, blueberry, blackberry, and cherry) are the preferred hosts, but some grape varieties have been reported as susceptible (Kenis et al., 2016). Unlike other Drosophilidae, SWD penetrates the skin of undamaged ripening fruits using its sclerotized serrated ovipositor and damage occurs due to subsequent larval feeding, but also via bacterial contamination and the rapid rotting of the fruit. SWD has caused huge financial losses in the newly invaded areas (Rota-Stabelli et al., 2013). The estimated annual costs to US fruit production are more than US $ 500 m. In addition, in the Trento district of Italy, the annual losses of small fruit production have been estimated as € 3.3 m per year (Bolda et al., 2010). Understanding diapause is essential for pest control by predicting the occurrence of economically important insects. Adult reproductive diapause is a powerful overwintering strategy for many continental insect species including Drosophila, it enables females to survive several months through harsh winter conditions and then lay eggs when the temperature and photoperiod increased (Zhai et al., 2016). Several studies have shown that SWD adults collected in the autumn were reproductively immature, however, there are different points on diapause in SWD. Toxopeus et al. suggested that the delayed reproductive maturity of winter-acclimated SWD appears to be temperature dependent, and is thus unlikely to be “true” diapause (Toxopeus et al., 2016). However, recently some field and laboratory studies suggested that the occurrence of diapause in overwintering reproductive adults (Rossi-Stacconi et al., 2016; Shearer et al., 2016; Wang et al., 2016). In addition, diapause has been studied in many drosophilids (e.g., *D. robusta*, *D. littoralis*, *D. montana*, *D. triauraria*, and *D. melanogaster*) (Carson and Stalker, 1948; Lumme et al., 1973; Yamada and Yamamoto, 2011; Salminen and Hoikkala, 2013; Kubrak et al., 2014). However, to the best of our knowledge there have been no specific studies of the mechanisms that affect female reproductive diapause in SWD.

Previous studies have not investigated the molecular mechanisms that regulate diapause in adult reproductive SWD females. In this study, we studied diapause and non-diapause populations of SWD to analyze the differentially expressed genes/proteins using proteomic and transcriptomic approaches. The results suggested that genes/proteins related to the citrate cycle, insulin signaling pathway, and target of rapamycin (TOR) signaling pathway may play important roles in adult reproductive diapause. This is the first study to investigate the molecular mechanisms responsible for regulating diapause in adult reproductive SWD females, and our results many facilitate the development of a fundamental understanding of reproductive diapause in economically important pest insects.

## Experimental Section

### Insect Rearing

A field population of SWD was collected from cherry orchards at Tai’an (35°67′N, 116°24′E) in Shandong Province, China during 2012. The strain was reared by continuous laboratory culture on artificial medium. The insects were maintained in the laboratory at 25 ± 1°C under a 16:8 h light: dark cycle. Female flies were collected within 12 h after eclosion and reared on artificial medium at 10 ± 1°C for 15 days under a long-day length photoperiod (16L: 8D) to generate non-diapause females and under a short-day length photoperiod (8L: 16D) to generate diapause females (Zhai et al., 2016). The principal criterion for reproductive diapause in fruit flies is the developmental status of the ovaries (King, 1971). Two biological replicate samples were stored at −80°C until use, where each sample was divided into two parts for global proteomics and whole-genome transcriptomics analyses.

### Protein Extraction and Trypsin Digestion

Samples from non-diapause and diapause females were ground into a powder in liquid nitrogen, homogenized in lysis buffer (8 M urea, 1% Triton-100, 65 mM DTT and 0.1% Protease Inhibitor Cocktail III), and then centrifuged at 12,000 rpm at 4°C. The supernatant was precipitated with cold 15% trichloroacetic acid/acetone for 2 h at −20°C. After centrifugation at 12,000 rpm at 4°C for 10 min, the remaining precipitate was washed with cold acetone three times, and the protein was then redissolved in buffer (8 M urea, 100 mM TEAB, pH 8.0), before the protein concentration was determined using a 2-D Quant kit (GE Healthcare, United States).

The total protein (100 μg) solution was reduced with 10 mM DTT for 1 h at 37°C and alkylated with 20 mM IAA for 45 min at room temperature in the dark. The proteins were then diluted by adding 100 mM TEAB to obtain a urea concentration of less than 2 M. Finally, trypsin was added at a trypsin: protein mass ration of 1:50 for the first digestion overnight and 1:100 for a second digestion of 4 h.

### TMT Labeling and LC-MS/MS Analysis

After trypsin digestion, the peptides were desalted using a Strata X C18 SPE column (Phenomenex) and vacuum-dried. The peptides were processed according to the manufacturer’s protocol using 6-plex TMT kit and labeled as follows: diapause female-1 (DF1), 128; diapause female-2 (DF2), 129; non-diapause female-1 (NF1), 130; and non-diapause female-2 (NF2), 131. The peptide mixtures were then incubated for 2 h at room temperature and pooled, before desalting and drying by vacuum centrifugation.

The peptides were subjected to an NSI source followed by tandem mass spectrometry (MS/MS) with a Q Exactive^TM^ Plus (Thermo Fisher Scientific) coupled online to the UPLC. Intact peptides were detected in the Orbitrap at a resolution of 70,000. Peptides were selected for MS/MS using an NCE setting of 30 and ion fragments were detected in the Orbitrap at a resolution of 17,500. A data-dependent procedure that alternated between one MS scan followed by 20 MS/MS scans was applied to the top 20 precursor ions above a threshold ion count of 2E4 in the MS survey scan with dynamic exclusion for 30.0 s. The electrospray voltage applied was 2.0 kV. Automatic gain control was used to prevent overfilling of the ion trap and 5E4 ions were accumulated to generate the MS/MS spectra. The m/z scan range was 350–1,800 for the MS scans. The fixed first mass was set to 100 m/z.

### Database Searches

All of the MS/MS data were processed using the Mascot search engine (v.2.3.0) with the target-decoy database search strategy (Elias and Gygi, 2007) against the *D.suzukii_OGS10_proteins.fasta* database. Trypsin/P was specified as the cleavage enzyme and we allowed up to two missing cleavages. The mass error was set to 10 ppm for precursor ions and 0.02 Da for fragment ions. Carbamidomethylation on Cys, TMT-6plex (N-term) and TMT-6plex (K) were specified as fixed modifications, and oxidation on Met was specified as a variable modification. The false discovery rate (FDR) was adjusted to <0.01 and the peptide ion score was set to >20. At leaset 2 unique peptides were used for the protein quantification, and *t*-test *p*-value among different unique peptides lower than 0.05 were considered to be significant (Chen et al., 2016).

### RNA Isolation, Quality Controls, and RNA Sequencing

Total RNA was extracted using an E.Z.N.A.^®^ Total RNA Kit II (Omega, United States) according to the manufacturer’s protocol. The samples were treated with DNase, and the quantity and quality of each RNA sample were assessed using a microvolume spectrophotometer (NanoDrop 2000, Thermo Fisher Scientific) and Bioanalyzer (Aglient 2100, Life Tech). Only the RNA samples with 260:230 ratios from 2.0 to 2.5, 260:280 ratios from 1.9 to 2.1, and RNA integrity numbers over 8.0 were used in the analysis.

Construction of the cDNA libraries and RNA-Seq were performed by the Biomarker Biotechnology Corporation (Beijing, China). According to the Illumina manufacturer’s instructions, Poly(A)^+^ RNA was purified from 10 μg of the pooled total RNA using oligo(dT) magnetic beads and fragmented into short sequences in the presence of fragmentation buffer. The cleaved mRNA was transcribed with random hexamers, and second-strand cDNA synthesis was performed. After purifying cDNA using AMPure XP (Beckman Coulter, United States) beads, endrepair and ligation of the adaptors, the products were amplified by PCR to create a cDNA library. Each cDNA library was sequenced using the Illumina sequencing platform (Hiseq 2500).

### Bioinformatics Analysis

Reads sequenced from each sample were aligned with the UniGene library using Bowtie (Langmead et al., 2009). To obtain the relative expression levels in each sample, the fragments per kilobase of transcript per million mapped reads (FPKM) in each sample were counted and combined with RSEM (Li and Colin, 2011). To ensure the reliability of the differentially expressed genes (DEGs), Pearson’s Correlation Coefficient (r) was used as an indicator to evaluate the correlation between two biological replicates. The DEGs were identified using the DESeq package with the Benjamini-Hochberg procedure. The global FDR < 0.01 and a fold change ≥2 in the FPKM value in two comparison groups were used as the thresholds to determine significant differences in the gene expression level.

### Western Blot Analysis

Modified western blot analyses were performed according to previously described methods (Zhai et al., 2013). The proteins were separated on a 12% SDS-PAGE gel and transferred to PVDF membranes (0.4 μm, Millipore), before the membranes were immunoblotted using the following antibodies: anti-FoxO (Forkhead box protein O, ab195977), anti-Hsp70 (Heat shock 70 kDa protein, ab2787), and anti-α-Tubulin (ab52866) from Abcam. Anti-FKBP12 (FK506-binding protein 12), anti-JHAMT (Juvenile hormone acid O-methyltransferase), and anti-YP1 (yolk protein 1) polyclonal sera were prepared in our laboratory. IgG goat anti-rabbit and anti-mouse antibodies conjugated with HRP were used as secondary antibodies (1:5,000, Abcam, United Kingdom), and the membranes were visualized by ECL (enhanced chemiluminescence). Three biological replicates were performed for each protein.

### Quantitative Real-Time PCR Analysis

The primers used for real-time PCR are listed in Supplementary Table S1. The synthesized first-strand cDNA was amplified by PCR in 10 μL reaction mixtures using a Light Cycler 480 system (Roche, United States) and *α-Tubulin* was used as the internal control gene (Zhai et al., 2014). After PCR amplification, melting curve analysis was performed in triplicate and the results were averaged. The quantitative variation was calculated using three independent biological samples with the relative quantitative method (2^−ΔΔCT^).

### Quantitative Determination of Hormone

JH III analyses were modified from methods described previously (Zhou et al., 2011). *Drosophila suzukii* samples were separately ground in grinder and ultra sonicated with methanol and isooctane. After centrifugation, the upper layer was transferred into a test tube. The residue was reconstituted in methanol, analyzed using HPLC-MS/MS (Agilent 6420; Waldbronn, Germany). JH III was separated using gradient elution and the hormone titer was expressed as ng per mg body weight.

## Results

### Global Changes in the Protein Levels

**FIGURE 1:**
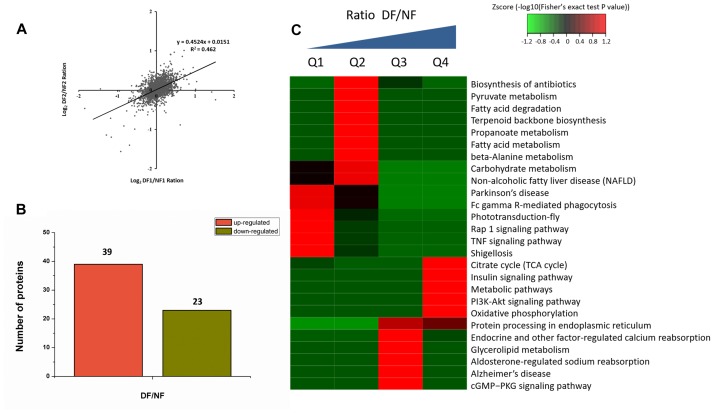
TMT analysis of the differentially expressed proteins data in diapause and non-diapause females. **(A)** The relative quantitative correlation of the two biological replicates of the proteome. **(B)** Number of differentially quantified proteins. On the basis of duplicate biological replications analyses, only proteins that changed ≥1.2-fold in relative ratios (*p* < 0.05) were considered. **(C)** Enrichment and clustering analysis of the quantitative proteomics data sets based on KEGG pathway database, and the quantitative proteins were divided into four groups (Q1–Q4); two groups for down-regulated (Q1: less than 1/1.2; Q2: 1/1.2 -1) and two groups for up-regulated (Q3: 1-1.2, Q4: more than 1.2). The color index (z-score) is showed in the legend, the red color represents the proteins that were significantly accumulated in KEGG pathway.

Proteins from the NF and DF were used for TMT labeling and HPLC fractionation, followed by high-resolution LC-MS/MS analysis and quantitative global proteome analysis. First, the MS data were validated, where the lengths of most of the peptides were between eight and 16 residues, which agreed with the properties of the tryptic peptides (Supplementary Figure S1). The mass error distribution was close to zero and most of the errors were less than 0.02 Da, so the mass accuracy of the MS data matched the requirements (Supplementary Figure S2). The relative quantitative correlations between the two biological replicates of the proteome were also acceptable (Figure 1A). Using the samples, 2,378 proteins were annotated based on the *D.suzukii_OGS10_proteins.fasta* database, and 2,375 proteins were quantified (Table 1). Using a quantification ratio of >1.2 as the up-regulated threshold and < 0.83 as the down-regulated threshold, 62 annotated proteins had statistically significant responses (*p* < 0.05). Among these annotated proteins, 39 and 23 proteins were up- and down-regulated, respectively (Figure 1B and Supplementary Table S2). To identify the cellular pathways regulated by diapause, we performed pathway clustering analysis for diapause based on pathways in the Kyoto Encyclope-dia of Genes and Genomes (KEGG). We found that some metabolic and signaling pathways, such as the citrate cycle, oxidative phosphorylation, insulin signaling pathway and PI3K-Akt signaling pathway, were the main pathways in quantiles with decreased protein levels in diapause females (Figure 1C).

**Table 1:** The high confidence quantitative proteome: size and features.

Identified nonredundant peptides	68,768
Identified nonredundant proteins	2,378
Quantifiable proteins (with unique peptides > 0 + non unique > 1)	2,375
Differential expressed proteins (*p*-value < 0.05, ratio ≥ 1.2 or ≤ 0.83)	62
Up-regulated proteins	39
Down-regulated proteins	23

**Table 2:** Statistics tables of clean reads mapped with reference genome.

Samples	Total reads	Mapped reads	Mapped ratio	Uniq mapped reads	Uniq mapped ratio
NF1	16,277,608	10,366,502	63.69%	9,119,638	56.03%
NF2	20,422,168	13,500,541	66.11%	11,800,695	57.78%
DF1	18,662,526	12,477,406	66.86%	10,813,386	57.94%
DF2	17,858,808	11,634,751	65.15%	10,411,901	58.30%

### Transcriptomic Analysis of DEGs

**FIGURE 2:**
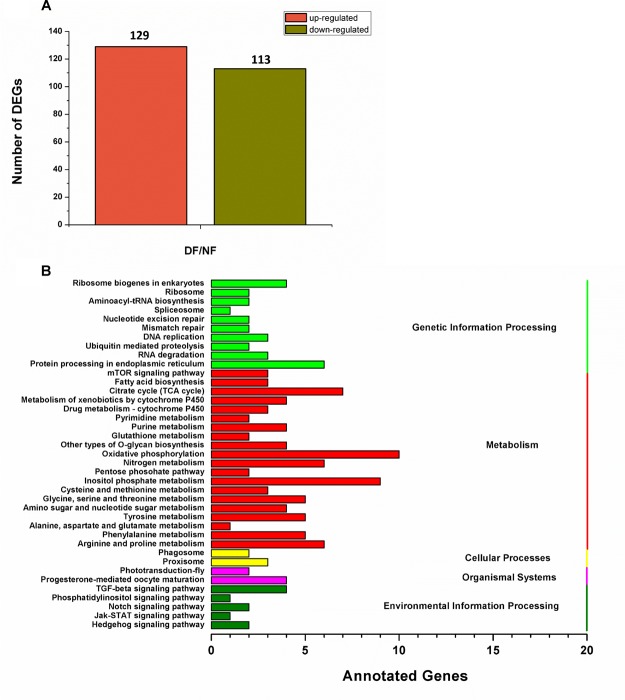
Transcriptomic analysis of the DEGs data in diapause and non-diapause females. **(A)** Number of significantly changed annotated DEGs, the conditions for genes was FDR ≤ 0.01 and FC ≥ 2. **(B)** The distribution of pathways of DEGs annotated in the KEGG data library.

In total, 73,221,110 clean single-end reads were generated by Illumina sequencing and mapped to the SWD reference genome. The mapped ratio of clean reads ranged from 63.69 to 66.86% (Table 2). The mapped density of reads on some chromosomes (Supplementary Figure S3) and the numbers in different regions of the reference genome are shown in Supplementary Figure S4A. Among 297 new genes without annotated functions, 259 were annotated based on NR (259), GO (208), COG (57), Swiss-Prot (163), and KEGG (87) (Supplementary Table S3). Sufficient and effective information was employed in this study because the saturation of the gene number increased with the number of sequenced reads (Supplementary Figure S4B). According to the gene expression level (FPKM), we analyzed the reliability of the DEGs by evaluating the correlations between two biological replicates in NF and DF (Supplementary Figure S5). Using a quantification ratio of >2 as the up-regulated threshold and < 0.5 as the down-regulated threshold, 242 annotated DEGs had statistically significant responses (*p* < 0.05). According to this analysis, among these annotated DEGs in non-diapause and diapause females, 129 and 113 genes were up- and down-regulated, respectively (Figure 2A). To identify pathways regulated by diapause treatment, we performed pathway clustering analysis based on the pathways in KEGG, which showed that most of the DEGs were correlated with metabolic processes, including the mTOR signaling pathway, citrate cycle, oxidative phosphorylation and amino acid metabolism (Figure 2B).

### Validation of DEGs and Proteins

**FIGURE 3:**
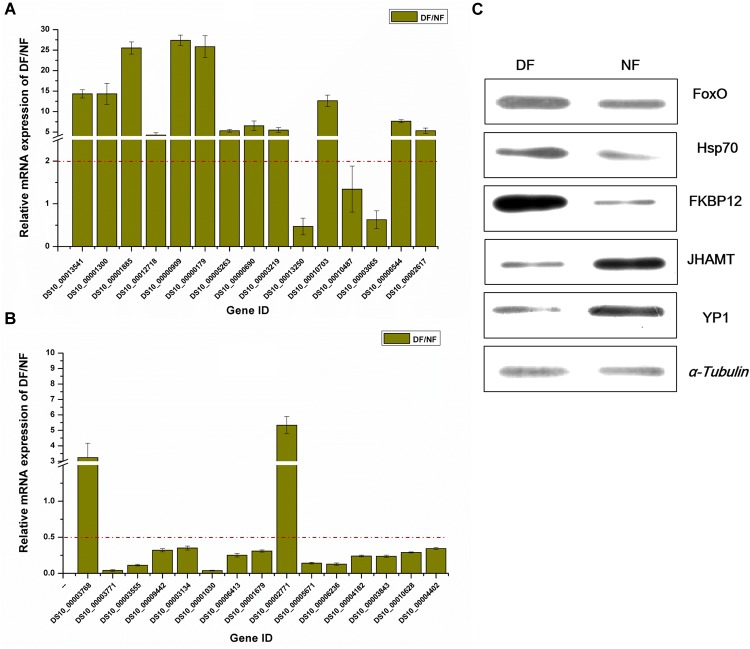
Validation of differentially expressed genes/proteins. Thirty DEGs and five proteins were selected. Fifteen up-regulated genes **(A)** and 15 down-regulated genes **(B)** by transcriptomic analysis, respectively. Three up-regulated proteins and two down-regulated proteins **(C)** represent the quantitative proteomic analysis results, respectively. The dark yellow bars represent the qRT-PCR results, presented as the mean ± SE (*n* = 3), gene ID form http://spottedwingflybase.oregonstate.edu/query. The proteins were separated on a 12% SDS-PAGE gel and immunoblotted with antibodies. *α-Tubulin* was used as an internal control, each protein sample was analyzed in triplicate **(C)**.

To further validate the gene and protein expression profiles, we selected 30 DEGs (15 up-regulated genes and 15 down-regulated genes) for qRT-PCR. In addition, five proteins were differentially expressed according to western blot analysis, i.e., three up-regulated proteins (FoxO, Hsp70, and FKBP12) and two down-regulated proteins (JHAMT and YP1). The results showed that 25 of the 30 DEGs were consistent with the results of the transcriptomic analysis, excluding DS10_00013250, DS10_00010487, DS10_00003065, DS10_00003768, and DS10_00002771 (Figure 3A,B). At the protein level, all five proteins differed in terms of their expression levels according to western blotting and proteomic analyses (Figure 3C).

### Correlations Between mRNA and Protein Expression Profiles

**FIGURE 4:**
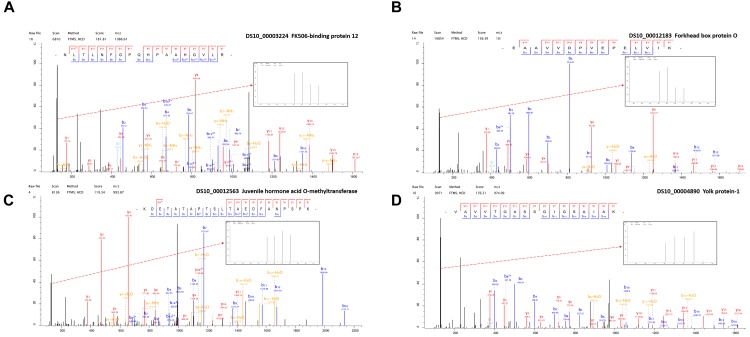
The representative MS/MS spectra of some differentially expressed proteins. **(A)** FK506-binding protein 12 kDa **(B)** Forkhead box protein O **(C)** Juvenile hormone acid O-methyltransferase **(D)** Yolk protein-1.

**FIGURE 5:**
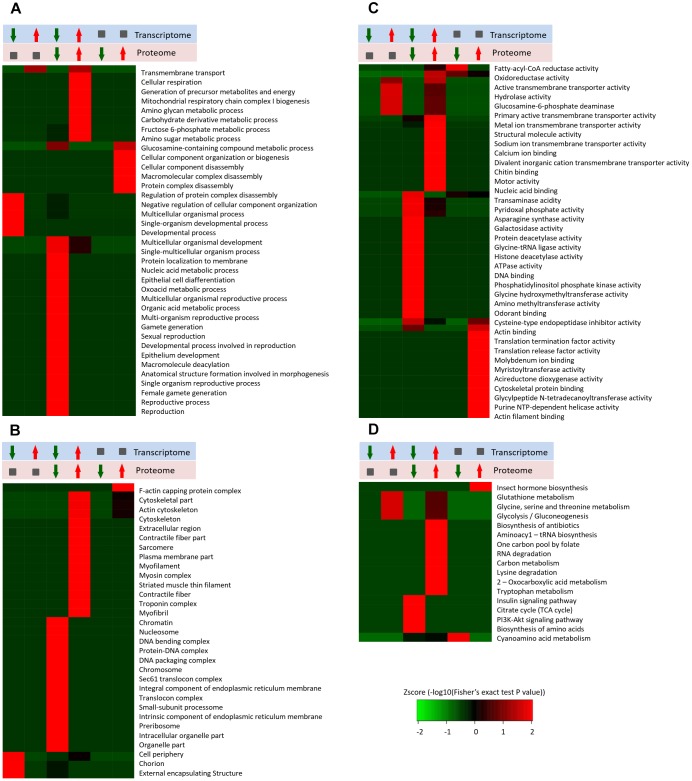
Enrichment and clustering analysis of the quantitative transcriptome and proteome data sets based on GO and KEGG annotations. Quantifiable genes and proteins were classified by gene ontology annotation based on three categories: **(A)** molecular function; **(B)** cellular compartment, and **(C)** biological process. Quantifiable proteins were annotated based on the KEGG pathway database **(D)**. These ratios were classified in six groups, red arrowhead represents up-regulated, green arrowhead represents down-regulated, gray square represents no changed. The *p*-values were transformed into z-scores prior to hierarchical clustering analysis.

In this study, we identified 2,375 quantifiable proteins, and 2,264 of the corresponding transcripts were quantifiable. In order to explore the relationships between the transcriptome and proteome in this study, we analyzed the relative quantitative correlations between the transcriptome and proteome (Supplementary Figure S6). We set a log2 quantification ratio of >0.585 as the up-regulated threshold and <-0.585 as the down-regulated threshold, and other rations were considered normal. Table 3 shows the relationship between the differentially expressed components of the transcriptome and proteome (Table 3). The overall analysis of robustly regulated genes at the protein and mRNA levels found four genes that overlapped in the up-up regulated group and six genes in the down-down regulated group, where glutathione S-transferase (GST), larval serum protein, FKBP12, and FoxO were up-up regulated proteins/genes, and 40S ribosomal protein, YP1, YP2, YP3, JHAMT, and NADH dehydrogenase were down-down regulated proteins/genes (Figure 4). To further validate the correlations between gene and protein expression profiles, we selected 4 up-up-regulated genes and 4 down-down-regulated genes for qRT-PCR, the 8 DEGs were all consistent with the results of the transcriptomic and proteomic analysis (Supplementary Figure S7). In order to further understand the biological functions of the differentially expressed proteins, we analyzed the quantifiable data set according to three enriched GO categories and KEGG pathways (Figure 5). In the down-down regulated group, some proteins/genes related to female gamete generation, multi-organism reproductive process, developmental process involved with reproduction, and related reproductive processes were significantly enriched in the biological process category (Figure 5A). In agreement, the analyses base on cellular compartment (Figure 5B) and molecular function (Figure 5C) showed that nucleic acid metabolism and energy metabolism proteins/genes were enriched in the two categories, respectively. Moreover, enrichment analysis based on KEGG (Figure 5D) showed that pathways involved with the citrate cycle, insulin signaling pathway, PI3K-Akt signaling pathway and biosynthesis of amino acids were enriched in the down-down regulated group.

**Table 3:** Differentially expressed protein or transcription.

Differentially expressed protein or transcription		Proteome		
		Up	Down	Normal
	Up	4	2	123
Transcriptome	Down	7	6	100
	Normal	28	15	2,090

## Discussion

Environmental conditions are not always suitable for survival and insects employ multiple strategies for adaptation. Adult reproductive diapause allows many female insects to survive harsh winters and to reproduce when the environmental conditions improve (Salminen and Hoikkala, 2013). In general, most Drosophila species are susceptible to cold, and they exhibit rapid cold-hardening and acclimation responses (MacMillan et al., 2009). SWD is mainly found in warm-temperate regions and it is considered that this species enters a winter reproductive diapause (Zerulla et al., 2015; Rossi-Stacconi et al., 2016; Shearer et al., 2016; Wang et al., 2016). The overwintering site is not clear but it is generally thought that SWD overwinters beneath leaf litter or in the built structures associated with orchards or other agricultural setting (Jakobs et al., 2015). In our previous study, we found that most of the females trapped in or around winter had immature ovaries and we trapped no adults in the winter. The ovarian development stages and oviposition rate were different at 10 ± 1°C. All of the females had immature ovaries under a short photoperiod (8L:16D) and the reproductive diapause phenomenon gradually weaken as the temperature increased or decreased. By contrast, the ovaries developed rapidly under a long photoperiod (16L: 8D) at different temperatures, where all of the females were developing or had developed ovaries, and the oviposition rate was 100% at temperatures above 10 ± 1 °C (Zhai et al., 2016). In addition, the ovaries developmental status after 15 days of diapause and non-diapause inducing condition (Supplementary Table S4). By extended the diapause-inducing time, the absence of vitelogenic oocytes occurs after 30 days under diapause-inducing conditions (Supplementary Figure S8).

In *D. melanogaster*, five major genes are known in the central components of the circadian clock: *period*, *timeless*, *Clock*, *cycle*, *and cryptochrome* (Hardin, 2005). The *timeless and cryptochrome* were highly associated with diapause occurrence (Yamada and Yamamoto, 2011; Huang et al., 2015). Juvenile hormone (JH) regulates many physiological processes in insects, such as diapause, and JHAMT is considered to be critical for regulating JH synthesis (Denlinger and Tanaka, 1989; Shinoda and Itoyama, 2003). To further validate the circadian genes and JH related genes expression under different photoperiods, we selected *DS-timeless* and *DS-JHAMT* genes for qRT-PCR. We found the clock gene *DS-timeless* was up-regulated under DF conditions (Supplementary Figure S9). However *DS-JHAMT* gene was down-regulated, and the JH titer indicated that the hormone levels were significantly decreased under DF conditions (Supplementary Figures S9, S10). According to the results obtained in the present study and other studies (Rossi-Stacconi et al., 2016; Wang et al., 2016), we propose that the adult is the diapause overwintering stage.

Large-scale omics data sets at the whole tissue levels can obtain multilayered pictures of regulatory processes, where RNA-Seq transcriptomic and quantitative proteomics are two powerful approaches for large-scale investigations of translational and post-translational regulated networks. Among the 2,375 differentially expressed proteins identified in this study, 39 and 23 proteins were up- and down-regulated, respectively (Supplementary Table S2). According to the Illumina sequencing results, we found 242 annotated genes related to adult reproductive diapause, including 129 up-regulated and 113 down-regulated genes (Figure 2A). These proteins and genes are potential targets for further functional studies. For example, the Hsp70s proteins are a family of conserved and ubiquitously expressed heat shock proteins, which are very highly up-regulated by multiple forms of stress, and they appeared to be either up-regulated or unaffected by diapause. Hsp70 proteins have been shown to be up-regulated in the embryonic, larval, pupal, and adult diapause (Rinehart et al., 2007; Lopez-Martinez and Denlinger, 2008). By contrast, some studies have shown that Hsp70 was not elicited by the induction of diapause (Tachibana et al., 2005; Rinehart et al., 2006). GST catalyzes the conjugation of the reduced form of glutathione to xenobiotic substrates for the purpose of detoxification, thereby protecting cells from oxidative stress-induced damage. High levels of GST mRNA and protein have been detected in diapausing embryos, larvae, and pupae (Lu and Xu, 2010; Tu et al., 2015). Elevated levels of GST in diapause-destined individuals are probably correlated with the ability to limit oxidative damage during the cold winter (Zhang et al., 2012). Our data showed that metabolic and signaling pathways were the most prominent pathways in diapause females (Figure 1C, 2D), such as energy metabolism, insulin signaling and amino acid metabolism pathways. The tricarboxylic acid cycle (TCA cycle) is essential for producing usable energy for many important biological molecules. In the cotton bollworm, *Helicoverpa armigera*, cross-talk between the brain and fat body as a regulator of diapause and the TCA cycle may be a checkpoint for regulating different forms of diapause (Xu et al., 2012). Insulin signaling has been implicated as a major regulator of diapause via its effects on metabolic suppression and growth control (Sim and Denlinger, 2013). In *D. melanogaster*, the insulin signaling system promotes adult reproductive diapause via FoxO phosphorylation in the PI3K/Akt pathway (Williams et al., 2006). The regulation of ovarian development by insulin signaling is not limited to *Drosophila* and it is also evident in the mosquito *Culex pipiens* (Sim et al., 2015).

In order to improve the credibility of data sets, the integration of multi-omic approaches has been applied in many fields (Dittmer et al., 2012). Based on the differential expression of target proteins/genes, we found four common up-regulated proteins/genes, i.e., GST (P20432), larval serum protein (Q24388), FKBP12 (P48375), and FoxO (B4G4S8). FKBP12 (Figure 4A) inhibits the activity of the TOR protein (Somarelli and Herrera, 2007). TOR is an atypical serine/threonine kinase in the phosphatidylinositol kinase-related kinase (PIKK) family and a major regulator of growth in eukaryotes (Zoncu et al., 2010). FoxO (Figure 4B) is a transcription factor that acts downstream of the insulin and JH signaling pathways, which is normally activated via the suppression of insulin signaling and it activated some distinct gene networks that contribute to the diapause phenotype (Dong et al., 2011; Sim and Denlinger, 2013; Sim et al., 2015). In *Aedes albopictus*, FoxO may be involved with increasing the transcript levels of genes related to fatty acid synthesis, as observed in early diapause females (Poelchau et al., 2011). In addition, we identified six common down-regulated proteins/genes comprising 40S ribosomal protein (Q9VFE4), JHAMT (Q9VJK8), YP1 (P06607), YP2 (P02843), YP3 (P02844), and NADH (Q9V4E0) (Supplementary Table S2). JHAMT (Figure 4C) is the ultimate enzyme in the JH biosynthetic pathway, and it is considered to be critical for regulating JH synthesis (Shinoda and Itoyama, 2003). JH regulates many physiological processes in insects, such as diapause (Denlinger and Tanaka, 1989). Indeed, the diapause phenotype can be reversed via the application of a JH analog, and it has been suggested that the insulin signaling pathway may be involved with JH synthesis (Tatar et al., 2011), which may promote the development of oocytes and the synthesis and deposition of YPs (Riddiford, 2012). YPs (Figure 4D) provide essential nutrients during embryo development. In most insects, the fecundity of adult females is regulated primarily by the synthesis of YPs (Saunders et al., 1990). In our study, we used KEGG pathway classifications to analyze the combined TMT proteomic and RNA-Seq transcriptomic data, which showed that the citrate cycle, insulin signaling pathway, and TOR signaling pathway were decreased in response to the diapause treatment (Figure 5D). In addition, the FoxO and TOR pathways are both linked to insulin signaling, where they may be associated with integrating metabolic and growth responses (Sim and Denlinger, 2013).

**FIGURE 6:**
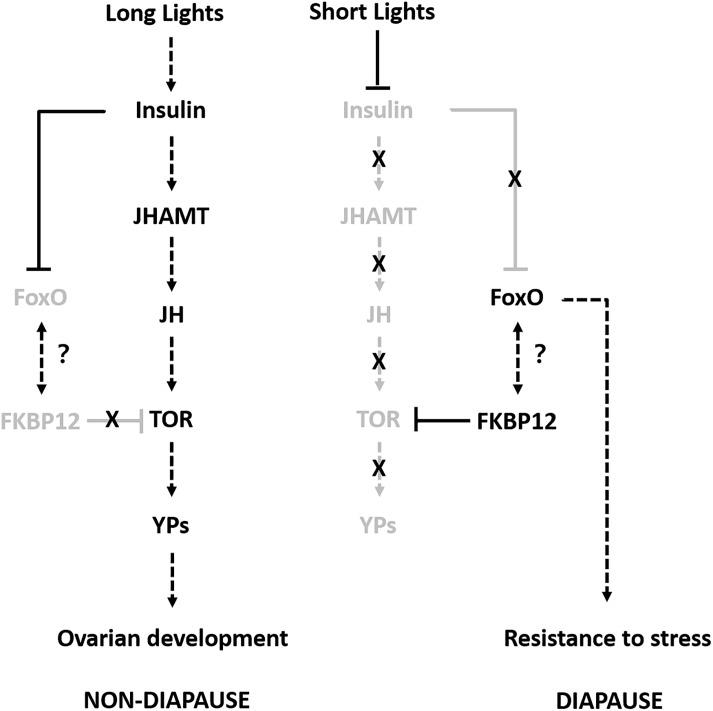
Proposed model for adult reproductive diapause regulation in SWD. Arrows indicate stimulation and T-bars indicate suppression. Black and gray indicate the ON and OFF activity states of the genes, respectively.

To the best of our knowledge, this is the first study to investigate the molecular regulatory mechanism responsible for the adult reproductive diapause in SWD. We combined multi-omics data in order to identify and compare the proteins related to adult reproductive diapause in SWD. The integrated comparison of mRNA and protein abundances indicated extensive translational and post-translational regulation. Therefore, we propose a possible model to explain how different photoperiodic signals might regulate the adult reproductive diapause in SWD (Figure 6). Some previous studies have suggested that many diapause candidate genes are compatible with flies younger than their chronological age, and they do not necessarily play roles in reproductive diapause and adaptation to environmental conditions (Reis et al., 2015). Thus, in future studies, we aim to determine the molecular functions of the common differentially expressed proteins/genes using the CRISPR/Cas9 system and provide suitable information to facilitate pest control.

## Ethics Statement

The SWD is an economically important pest insect in the world, which attacks a wide range of soft berries and stone fruits. The field studies did not involve endangered or protected species, and no specific permissions were required for our research activities in these locations.

## Author Contributions

YZ, XD, YY, and LZ conceived and designed the experiments. YZ, XD, HG, HC, and ZY preformed the experiments. YZ, PY, and PL analyzed the data and wrote the manuscript. All authors read and approved the final manuscript.

## Conflict of Interest Statement

The authors declare that the research was conducted in the absence of any commercial or financial relationships that could be construed as a potential conflict of interest.
