# Supplementary material for: Quantitative Proteomic and Transcriptomic Analyses of Metabolic Regulation of Adult Reproductive Diapause in Drosophila suzukii (Diptera: Drosophilidae) Females
Source: Front Physiol. 2019 Apr 4;10:344. doi: 10.3389/fphys.2019.00344 (PMC6458243; doi:10.3389/fphys.2019.00344)
Supplement: Supplementary file 1 [file Data_Sheet_1.docx]

**
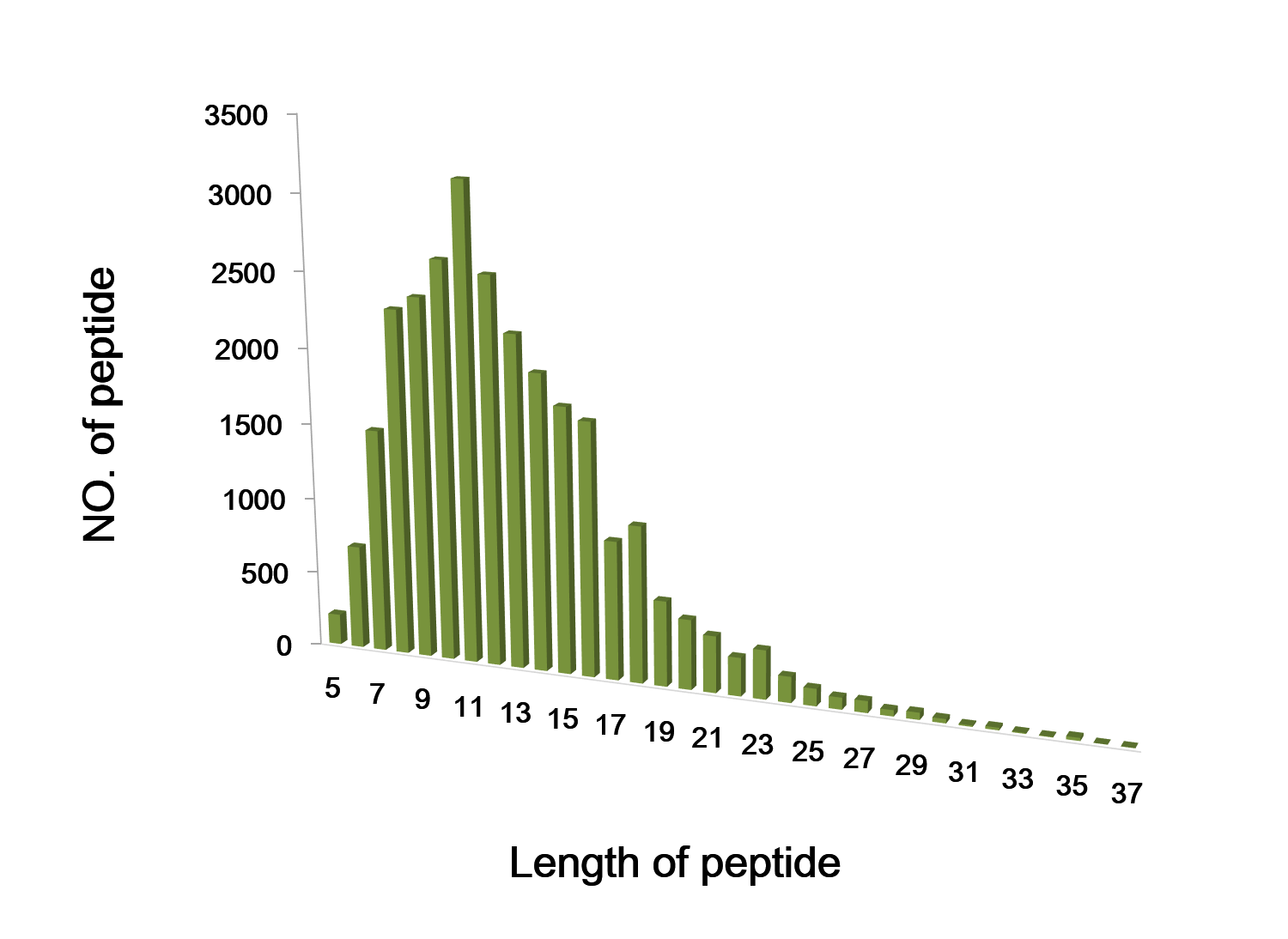
**

**Supplementary Figure 1.** Quality control validation of MS data, peptides length distribution.

**
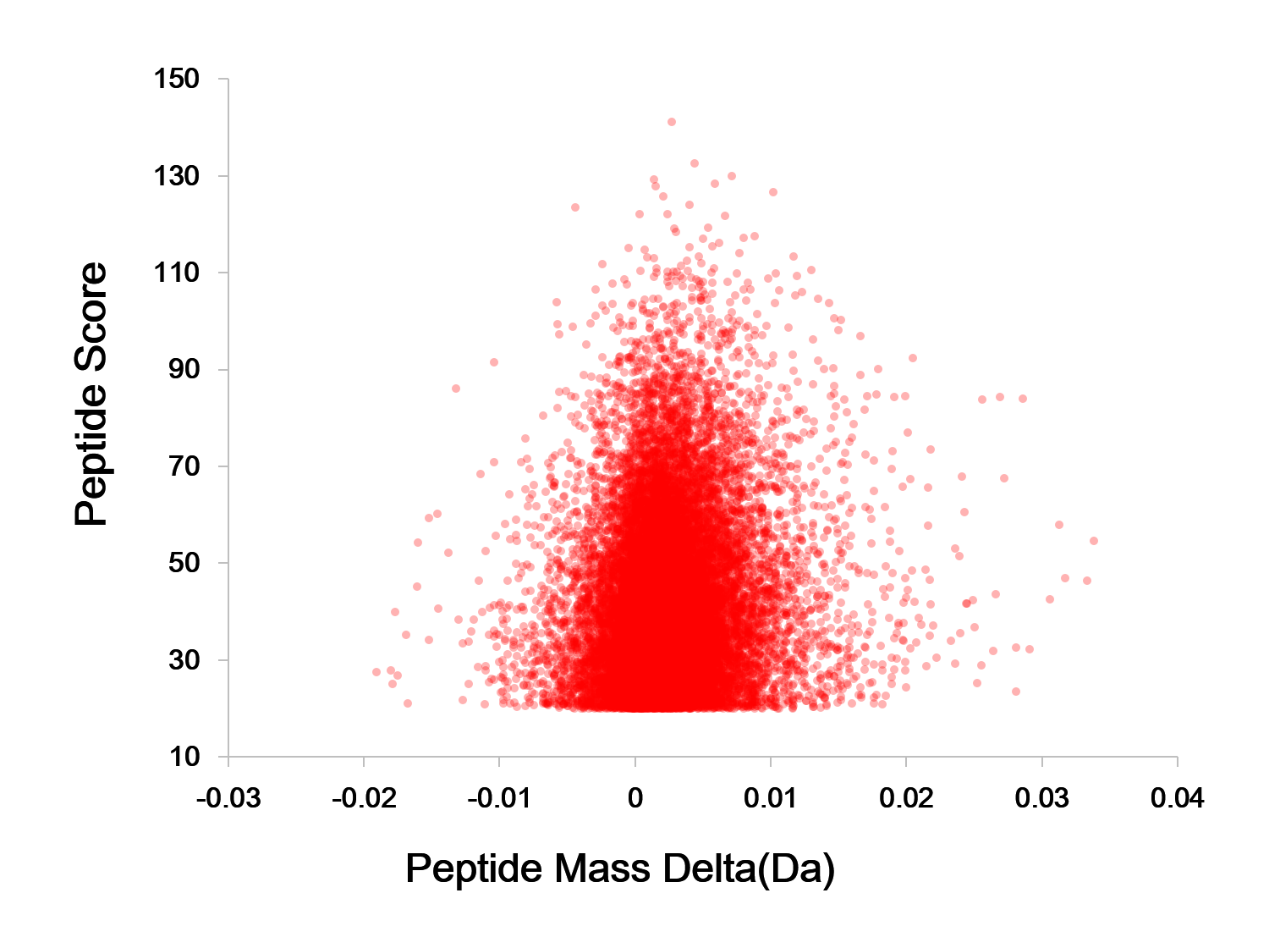
**

**Supplementary Figure 2.** Quality control validation of MS data. Mass error distribution of all identified peptides.

**
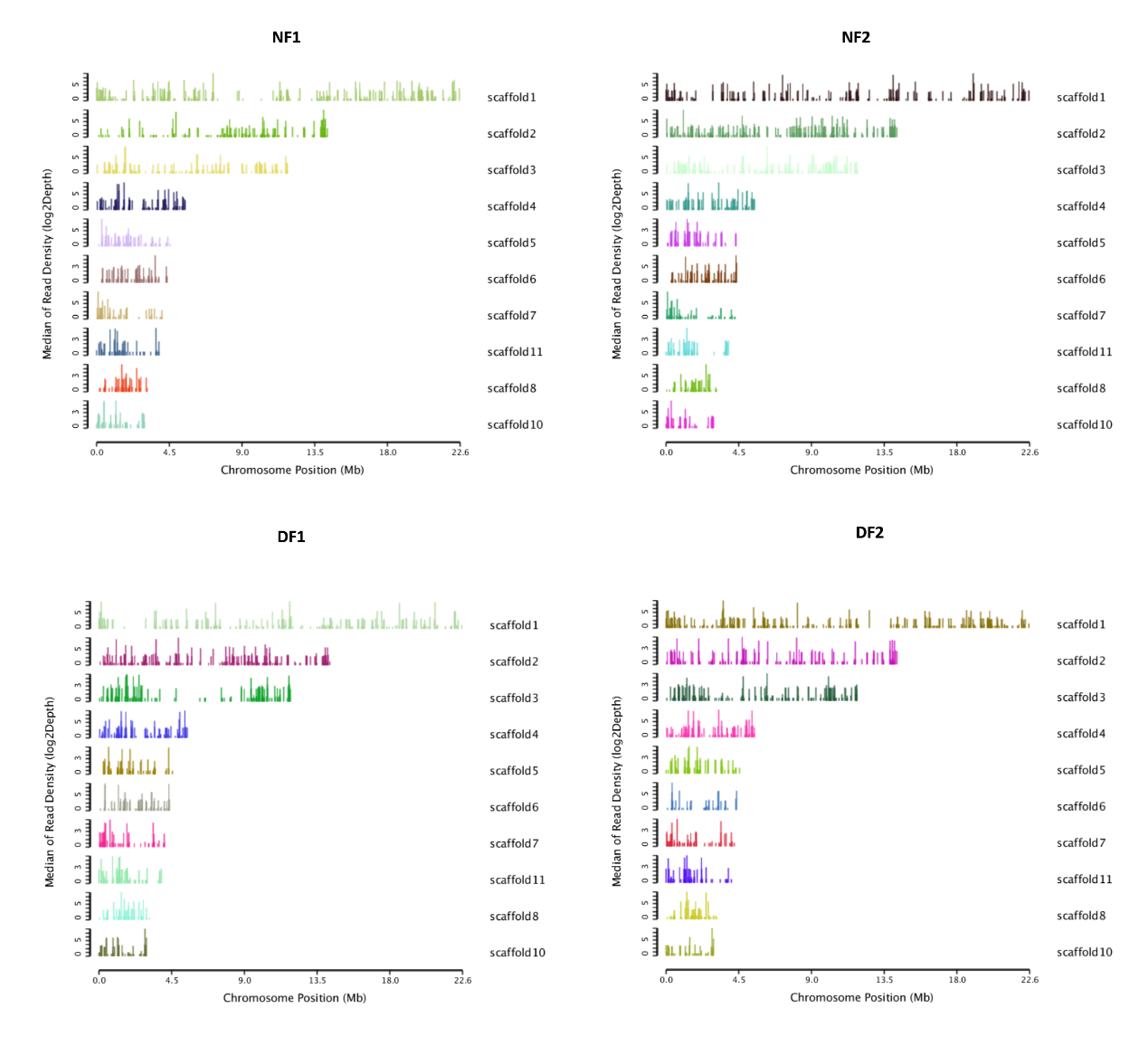
Supplementary Figure 3.** The mapped reads density on some chromosomes.

**
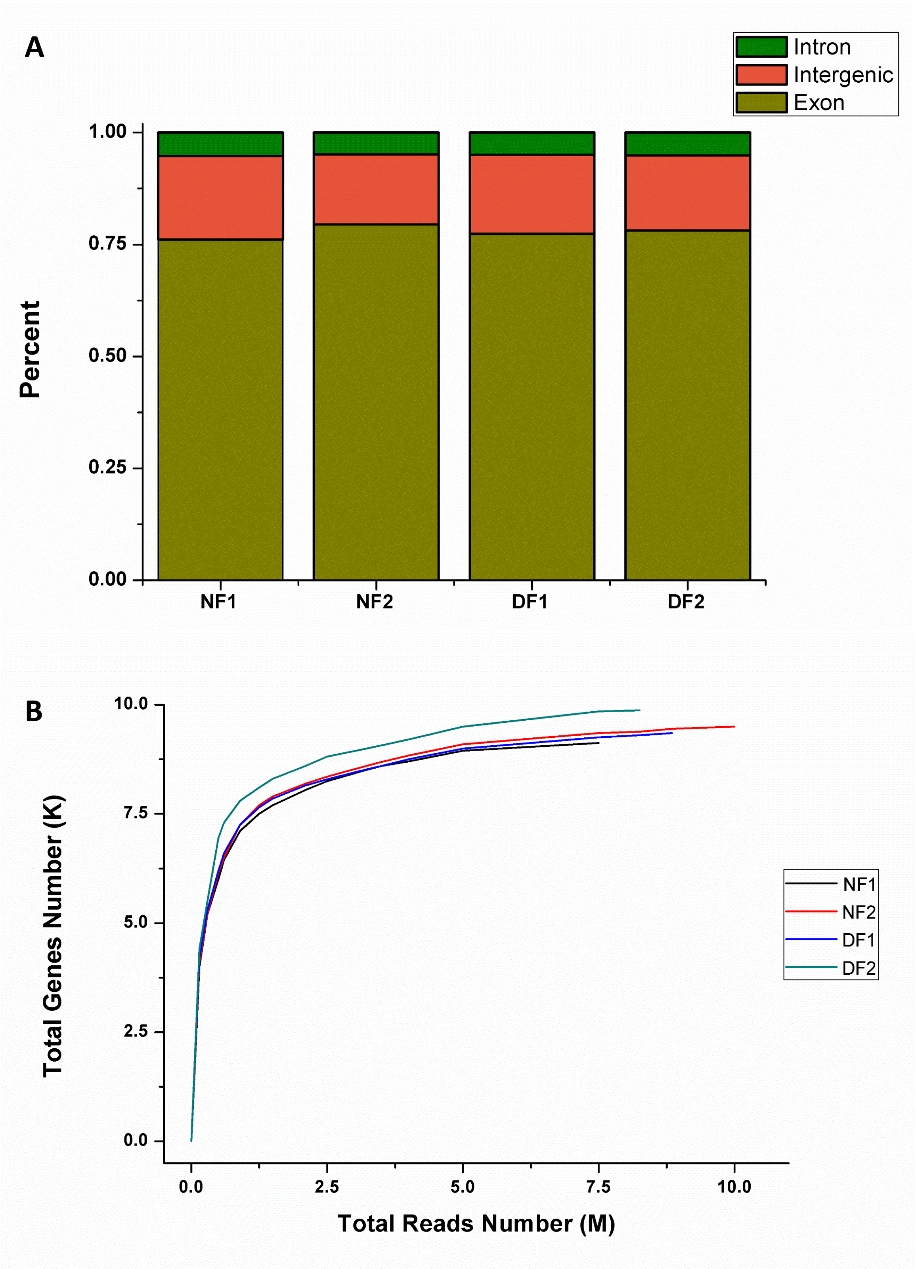
**

**Supplementary Figure 4.** Transcriptomic analysis of the DEGs data. (A) Genomic regions of reads distribution histogram. (B) Simulated diagram of saturation test of sequencing data. X axis indicates the number of reads (10^6^), Y axis indicates the number of detected genes (10^3^) and FPKM ≥ 0.1.

**
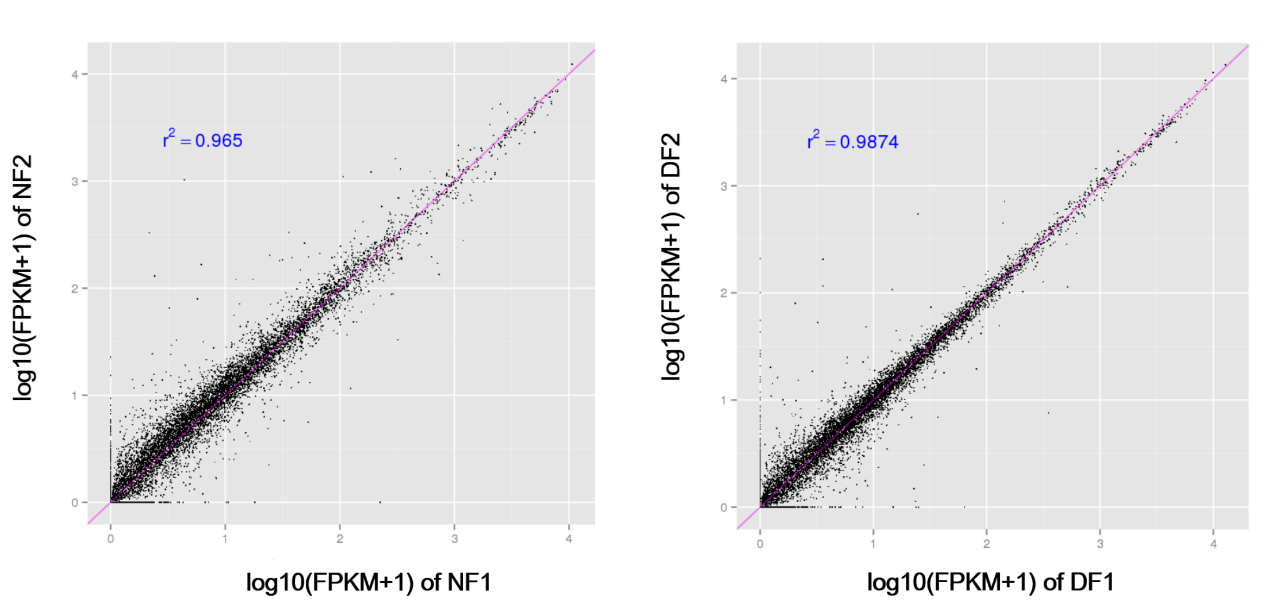
Supplementary Figure 5.** Correlation plot diagram of expression levels for unigenes of two biological replicates. Horizontal axis and vertical axis indicate the value calculated according to log (FPKM +1) of two biological replicates.

**

Supplementary Figure 6.** The relative quantitative correlation of transcriptome and proteome





**Supplementary Figure 7. Validation of diﬀerentially expressed genes/ proteins**


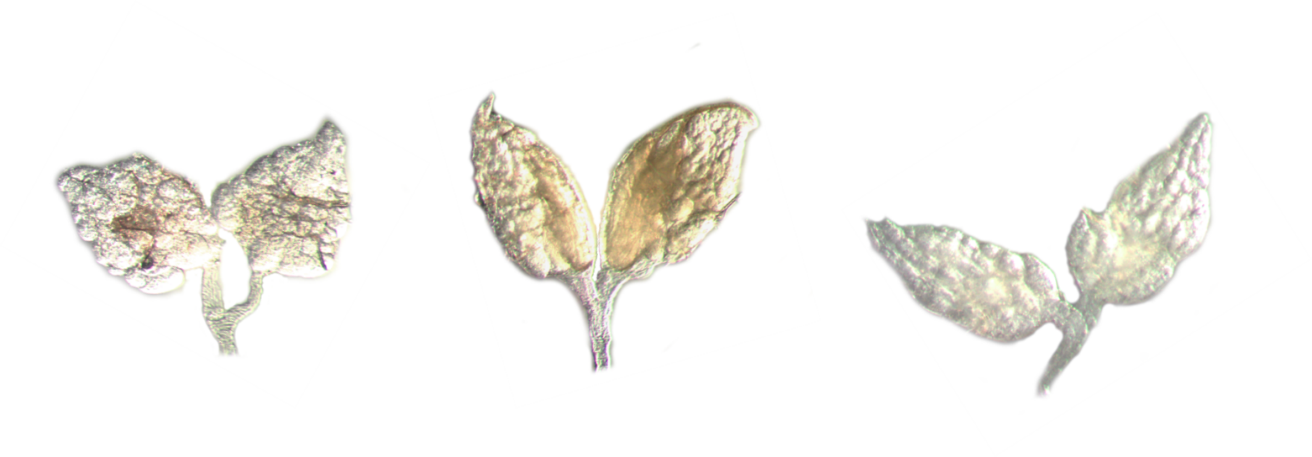


**Supplementary Figure 8.** The representative ovaries dissection figures after 30 days under diapause-inducing conditions. 35 individuals were tested for each of the treatments, and the experiment was replicated three times.





**Supplementary Figure 9. The relative mRNA expression of DF/NF.**


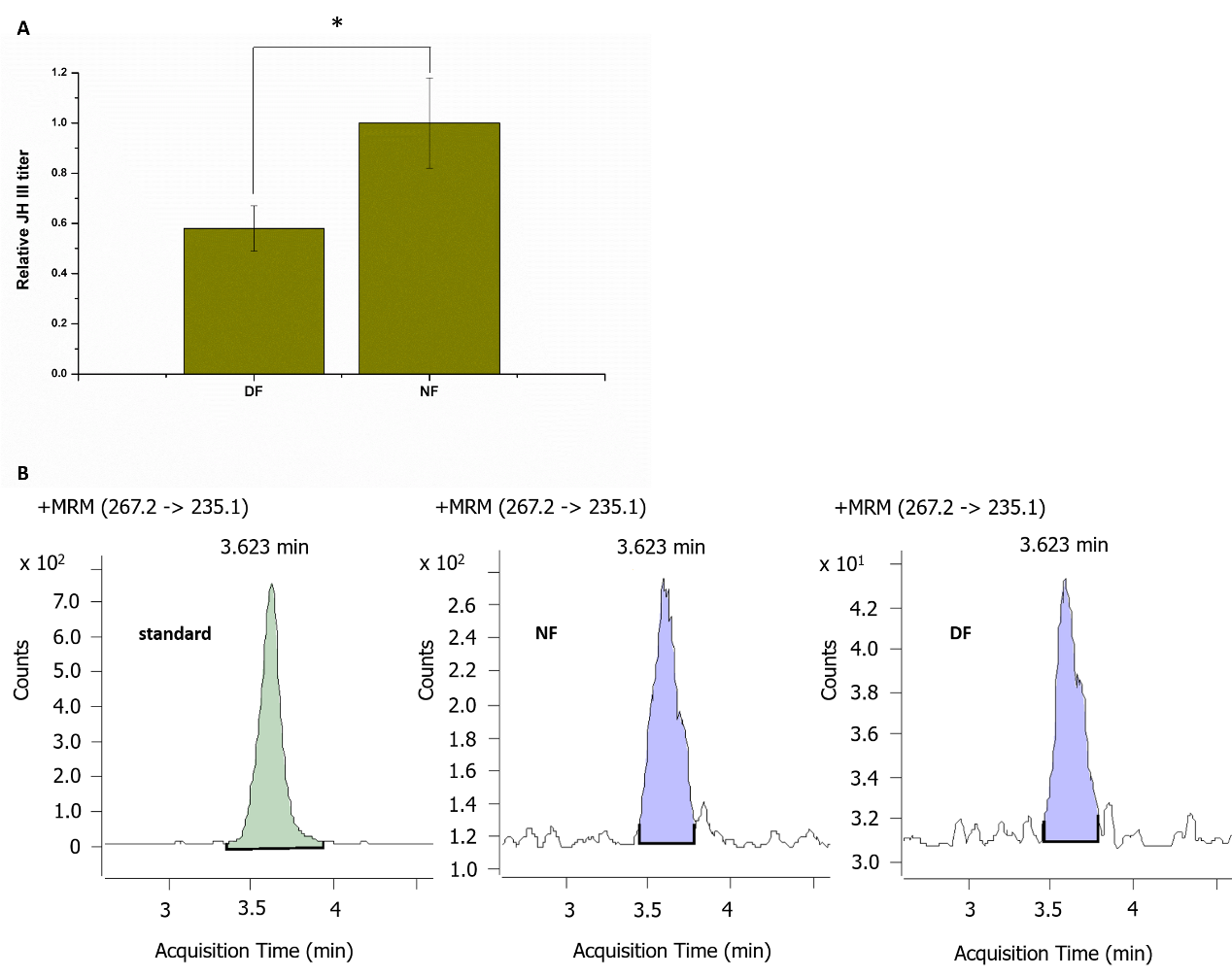


**Supplementary Figure 10.** (A)The JH III titer were analyzed at diapause and non-diapause status. (B) The representative chromatograms of JH III. Three replicates were conducted, with the data presented as mean ± SEM. Significant differences between treatment and control are indicated with asterisks (∗p < 0.05)
