# Supplementary material for: Quantitative Proteomic and Transcriptomic Analyses of Metabolic Regulation of Adult Reproductive Diapause in Drosophila suzukii (Diptera: Drosophilidae) Females
Source: Front Physiol. 2019 Apr 4;10:344. doi: 10.3389/fphys.2019.00344 (PMC6458243; doi:10.3389/fphys.2019.00344)
Supplement: Supplementary file 2 [file Data_Sheet_2.docx]

**Supplementary Table 1 Primer sequence of genes**

| Gene ID | Primer sequence(5'- 3') | |
| --- | --- | --- |
|  | **Forward** | **Reverse** |
| *DS10_00013541* | GAGCAGTTCGTTGAGGGAG | CGTGATGGGAGCCAGTAGA |
| *DS10_00001300* | CCTGATCCCAGTCCAACG | GCTGATAGGGCCACGAAG |
| *DS10_00001885* | GGACGCACCTGGCGATAA | CATTGCTCCCGCTCTTGG |
| *DS10_00012718* | GCGGTGGTGGAGCTGGTAA | CTGGTGGTTGATTCTTCTGTGG |
| *DS10_00000909* | GGCTCCCATCGCCATTAC | TCCGCCTCCGCAAACACT |
| *DS10_00000179* | AAGCCAAGGAAGAAGTCGG | CACAAAGTTGAGGTAGCCATTA |
| *DS10_00005263* | GGCCATCGCCAACTACGA | GGAAGCATTGCCACCCAC |
| *DS10_00000690* | CAACAATTTCCCTGTCTG | ATTGGAGCCGATCTTTAT |
| *DS10_00003219* | TGCTATTCAGCGGCTTCG | GCCACTCGCACATTCTCC |
| *DS10_00013250* | GTTTATCGCCTTCGTCTCG | GATCCTTCGCACCACTCG |
| *DS10_00010703* | TGGTTCAAGGCGACATTC | CTTTGGACACCCGACGAT |
| *DS10_00010487* | GACAGCGACGACAACAACT | GGAAGACATCCCAACCCT |
| *DS10_00003065* | ACGGAGTTGATAATCGTGTTTC | TCTCAGCACTGGCACTTGTT |
| *DS10_00006544* | TCGCTGCCATCAGTGCCT | GGGTCTTGGGTTCCGAGTAG |
| *DS10_00002617* | ACAATCGTGGTGAAAGCC | GGTGCTGATGGTGGTGTT |
| *DS10_00003768* | GGGTGGCTATGCCTACCAGG | CCAACATCAACGCCGCTAT |
| *DS10_00003771* | CCTGCTGCGTTGCTTTGA | TCCAGTCGGCACCGTTCA |
| *DS10_00003555* | AAGTGCGTTTGCTCCTCAT | GTGCCCTCGTATCCATTCT |
| *DS10_00009442* | AACCGAGAAACAATCCAGT | CAAGATGAGGGCGACAAA |
| *DS10_00003134* | TTGCCTTCCACTTGGCTTAT | TCCCTGTGCTGGTCGTCT |
| *DS10_00001030* | GCAGTCAGCGAGGAGACA | AGATGTTCAAGAGGGTGGTC |
| *DS10_00006413* | ACGCCATTACCTCTAAAC | GTGGTGTCTTCCTCCTCT |
| *DS10_00001679* | TCGTCGCTGCCGATAAGG | CGAGACCGGGTTGTAGGATG |
| *DS10_00002771* | GCTATCGCCCTGGCTTTG | TGTGCTCCACCACCTCCTG |
| *DS10_00005671* | GAACATGCCCATCGTCTCC | CAACGGCTCCTACACCAA |
| *DS10_00006236* | AGGACACGGAGCATTTGAT | GAACACCTCGCACATTCG |
| *DS10_00004182* | ATCACCGTCGGGAAATCA | CGAAGGACCTGCCAAAGT |
| *DS10_00003843* | TACACGCTGCCCAAGGAT | TCCCGTCTGCTGGATTTG |
| *DS10_00010628* | GACGACGACGCTGATGAT | CAGGAGATGCTGGAGGTG |
| *DS10_00004402* | TGTCCTCTACATAGCCCTCAT | CGCAGATACTCCGATTTCA |
| *DS-GST* | AGTCGGGCGTCTTGTTTC | AGTCGGCGATGGTTATGC |
| *DS-LSP* | GGAAGATTATCAGGCACT | AAGAGTATTTCGCAGACC |
| *DS-FKBP12* | AGGGAGTCGCCCAGTTGA | CGAAGGTGAGGGTGGAGTT |
| *DS-FoxO* | ATAAATCTGGTCGCTTCT | TTCCCTTCATTAGGTGGT |
| *DS-40SRP* | CGAGGACTTCTTGGAGCG | ACGAACGACGGGATGTTG |
| *DS-YP1* | CTGCAGCAAGTCTACCACCTG | GCGCTGGATCATCTCGTTCAG |
| *DS-JHAMT* | GCATTATCAGCGGGAAGA | TCGGGTTTCAGAAGATTGTA |
| *DS-NADH* | ATAGCTGCTCCTCCAACA | TCGAATTTTACCTCCTCT |
| *DS-timeless* | TCGCATCTTTCAACAAGTCT | TCACAATAGCCCGTCTCC |
| *a-Tubulin* | AGGATGCGGCGAATAACT | CGGTGGATAGTCGCTCAA |

**Supplementary Table 2. Proteins identified by TMT and RP HPLC-MS/MS**

| No. | Gene ID^a^ | UniProtKB | Protein name^b^ | Ratio | P value | Interpro ID | DF / NF  Regulated |
| --- | --- | --- | --- | --- | --- | --- | --- |
| a1 | DS10_00004610 | P11997 | Larval serum protein 1 gamma | 2.0245 | 6.00E-17 | IPR000896;IPR005203;IPR005204;IPR014756;IPR008922;IPR013788; | Up |
| a2 | DS10_00013464 | P11996 | Larval serum protein 1 beta | 1.935 | 3.63305E-16 | IPR005203;IPR005204;IPR014756;IPR008922;IPR013788;IPR000896; | Up |
| a3 | DS10_00004039 | Q04691 | Fat-body protein 1 | 1.768 | 6.22481E-16 | IPR014756;IPR005204;IPR000896;I | Up |
| a4 | DS10_00012183 | B4G4S8 | Forkhead box protein O | 1.624 | 0.003301204 | IPR006170;IPR023316; | Up |
| a5 | DS10_00003707 | Q8IQA2 | Tumorous testis | 1.624 | 0.016237837 | IPR012677;IPR000504; | Up |
| a6 | DS10_00009174 | Q9VB96 | aldehyde dehydrogenase | 1.526 | 6.94076E-07 | IPR029510;IPR015590;IPR016161;IPR016163;IPR016160; | Up |
| a7 | DS10_00004146 | Q24388 | Larval serum protein 2 | 1.457 | 1.98228E-14 | IPR014756;IPR000896;IPR008922;IPR005203;IPR005204;IPR013788; | Up |
| a8 | DS10_00003747 | P20432 | Glutathione S-transferase D1 | 1.396 | 0.009934106 | IPR005442;IPR004045;IPR012336;IPR010987; | Up |
| a9 | DS10_00001733 | Q9V3Z9 | wing disc development | 1.394 | 0.02066805 | PR008922; IPR005203 | Up |
| a10 | DS10_00002628 | Q24372 | Lachesin | 1.373 | 0.012862454 | IPR013098;IPR013783;IPR007110;IPR003598;IPR003599; | Up |
| a11 | DS10_00007221 | P42281 | Acyl-CoA-binding protein homolog | 1.335 | 0.042870148 | IPR022408;IPR000582;IPR014352; | Up |
| a12 | DS10_00013074 | P29829 | G protein β-subunit | 1.326 | 1.11485E-05 | IPR019775;IPR020472;IPR001632;IPR001680;IPR017986;IPR015943; | Up |
| a13 | DS10_00003761 | Q9VSN3 | Cuticular protein 66D | 1.3205 | 0.02686371 | IPR000618; | Up |
| a14 | DS10_00012482 | Q9VHE4 | Carbon-nitrogen hydrolase | 1.313 | 0.044672968 | IPR003010; | Up |
| a15 | DS10_00004344 | Q9VPF3 | 4-hydroxyphenylpyruvate dioxygenase | 1.305 | 0.002236506 | IPR005956;IPR004360;IPR029068; | Up |
| a16 | DS10_00005851 | A1Z7K9 | PAB-dependent poly(A)-specific ribonuclease | 1.298 | 0.03004033 | IPR001164;IPR028889;IPR028881;IPR017986;IPR013520;IPR012337;IPR015943;IPR006055;IPR010432; | Up |
| a17 | DS10_00004743 | P12646 | Glucose-6-phosphate dehydrogenase | 1.298 | 0.016595597 | IPR016040;IPR001282;IPR022675;IPR022674;IPR019796; | Up |
| a18 | DS10_00007799 | P17276 | Phenylalanine 4-monooxygenase | 1.277 | 0.010084597 | IPR018301;IPR019774;IPR019773;IPR001273;IPR005961;IPR002912; | Up |
| a19 | DS10_00009142 | Q9VA32 | Cuticular protein 100A | 1.27 | 0.001181957 | IPR000618; | Up |
| a20 | DS10_00002372 | P48596 | GTP cyclohydrolase 1 | 1.2665 | 0.006177375 | IPR001474;IPR018234;IPR020602; | Up |
| a21 | DS10_00007996 | A9UNH0 | Extracellular region | 1.259 | 4.19216E-11 | IPR018195;IPR001156; | Up |
| a22 | DS10_00001732 | Q23839 | Heat shock 70 kDa protein | 1.256 | 0.001509353 | PR013320; IPR005725;IPR004100; | Up |
| a23 | DS10_00006619 | A1ZBB4 | Precatalytic spliceosome | 1.2545 | 0.007887376 | IPR003034;IPR027417;IPR001870;IPR003877;I | Up |
| a24 | DS10_00007687 | P48602 | ATPase 68kD subunit A isoform 1 | 1.252 | 0.024673849 | IPR022878;IPR024034;IPR000793;IPR027417;IPR020003;IPR000194; | Up |
| a25 | DS10_00012391 | Q7KNR7 | Photoreceptor dehydrogenase, isoform C | 1.244 | 0.027635033 | IPR016040;IPR008907;IPR020904;IPR002198;IPR002424; | Up |
| a26 | DS10_00013260 | Q9VA83 | Ferritin | 1.239 | 5.40552E-06 | IPR008331;IPR009078;IPR009040;IPR014034;IPR012347; | Up |
| a27 | DS10_00011843 | Q9VTZ6 | Phosphomannomutase | 1.236 | 0.032342643 | IPR023214;IPR006379;IPR005002; | Up |
| a28 | DS10_00007106 | Q7JMV3 | Calcium/calmodulin dependent protein kinase I | 1.235 | 0.03582551 | IPR002290;IPR000719;IPR011009;IPR017441;IPR008271; | Up |
| a29 | DS10_00006898 | Q9VZ34 | Uncharacterized protein | 1.2305 | 0.004142803 | IPR006214; | Up |
| a30 | DS10_00012896 | P20348 | Sex-regulated protein janus-A | 1.229 | 0.002779981 | IPR007702; | Up |
| a31 | DS10_00012493 | Q960B1 | Uncharacterized protein | 1.2205 | 0.027940461 | IPR024660;IPR016024;IPR011989; | Up |
| a32 | DS10_00003404 | Q9W1V7 | Metal ion binding | 1.212 | 0.020378498 | IPR015880;IPR007087; | Up |
| a33 | DS10_00001190 | Q9VJ74 | ATP binding | 1.2115 | 0.017329919 | IPR014014;IPR000629;IPR014001;IPR011545;IPR027417;IPR001650; | Up |
| a34 | DS10_00001075 | M9ND31 | Glucosamine-6-phosphate isomerase | 1.2115 | 0.003123969 | IPR006148;IPR004547;IPR018321; | Up |
| a35 | DS10_00004379 | Q9VZZ6 | acyl-CoA hydrolase | 1.2105 | 0.034745163 | IPR003736;IPR029069;IPR006683; | Up |
| a36 | DS10_00003224 | P48375 | FK506-binding protein 12 kDa | 1.207 | 0.003596107 | IPR001179; | Up |
| a37 | DS10_00009844 | Q8SXD5 | defense response to virus | 1.2055 | 0.010841624 | R002884;IPR022398;IPR013785; | Up |
| a38 | DS10_00010159 | O17445 | 60S ribosomal protein L15 | 1.205 | 0.023897451 | IPR012678;IPR020925;IPR000439;IPR024794; | Up |
| a39 | DS10_00008337 | P26016 | Furin-like protease 1 | 1.203 | 0.003775041 | IPR008979;IPR000209;IPR000322;IPR009020;IPR017853;IPR015500;IPR023827;IPR023828;IP | Up |
|  |  |  |  |  |  |  |  |
| b1 | DS10_00007099 | Q9V4E0 | NADH dehydrogenase | 0.832 | 0.008322376 | IPR022885;IPR014029;IPR029014;IPR001135; | Down |
| b2 | DS10_00011368 | P33438 | Glutactin | 0.8295 | 0.025126901 | IPR029058;IPR019819;IPR002018; | Down |
| b3 | DS10_00001953 | Q9VKF6 | V-type proton ATPase subunit | 0.825 | 0.04720505 | IPR026028;IPR002490; | Down |
| b4 | DS10_00005310 | Q9V3S0 | Cytochrome P450-4g1 | 0.819 | 3.22548E-07 | IPR002401;IPR017972;IPR001128; | Down |
| b5 | DS10_00006919 | Q9VZ19 | Oxidoreductase | 0.817 | 0.010435455 | IPR002347;IPR016040;IPR002198; | Down |
| b6 | DS10_00011618 | Q9VD51 | ATP-dependent RNA helicase pitchoune | 0.812 | 0.011104532 | IPR025313;IPR000488;IPR001650;IPR014001;IPR014014;IPR011029;IPR027417;IPR011545; | Down |
| b7 | DS10_00001499 | P09615 | Protein wingless | 0.808 | 0.00289077 | IPR005817;IPR018161; | Down |
| b8 | DS10_00012207 | Q9VHW0 | Carbon-nitrogen ligase | 0.8 | 0.011273819 | IPR000120;IPR023631; | Down |
| b9 | DS10_00008892 | P84051 | Histone H2A | 0.795 | 0.000648597 | IPR009072;IPR002119;IPR007125; | Down |
| b10 | DS10_00007362 | Q6NN09 | ATPase | 0.795 | 0.000292177 | IPR000454;IPR002379;IPR020537; | Down |
| b11 | DS10_00002163 | Q9W1K0 | Transmembrane protein 14 homolog | 0.7945 | 0.047926801 | IPR005349; | Down |
| b12 | DS10_00011223 | Q9VFE4 | 40S ribosomal protein S5b | 0.794 | 0.021093185 | IPR020606;IPR023798;IPR000235;IPR005716; | Down |
| b13 | DS10_00002925 | Q0GT46 | Serine-type endopeptidase | 0.7885 | 0.007832215 | IPR009003;IPR001254;IPR001314; | Down |
| b14 | DS10_00012165 | Q9V3L7 | Respiratory electron transport chain | 0.7875 | 0.035123469 | IPR019329; | Down |
| b15 | DS10_00006498 | Q9W5X1 | EGF-like domain | 0.771 | 0.005168761 | IPR013032;IPR000742; | Down |
| b16 | DS10_00011851 | Q9VTZ0 | Uncharacterized protein | 0.755 | 4.74866E-05 | IPR025761;IPR025762;IPR025768;IPR025609;IPR019050;IPR010920; | Down |
| b17 | DS10_00002018 | Q8SY61 | Odorant-binding protein 56d | 0.727 | 0.035770013 | IPR006170;IPR023316; | Down |
| b18 | DS10_00012563 | Q9VJK8 | Juvenile hormone acid O-methyltransferase | 0.601 | 0.016873511 | IPR000966; | Down |
| b19 | DS10_00000690 | M9NE79 | Uncharacterized protein | 0.526 | 0.003403454 | IPR016187;IPR016186;IPR001304; | Down |
| b20 | DS10_00003771 | P22977 | Chorion protein S16 | 0.46 | 0.018366873 | IPR008450; | Down |
| b21 | DS10_00008400 | P06607 | Yolk protein-3 | 0.438 | 1.8664E-19 | IPR029058;IPR013818; | Down |
| b22 | DS10_00004890 | P02843 | Yolk protein-1 | 0.404 | 9.0772E-07 | IPR029058;IPR013818; | Down |
| b23 | DS10_00004891 | P02844 | Yolk protein-2 | 0.313 | 4.60471E-07 | IPR029058;IPR013818; | Down |

^a^ Gene ID form <http://spottedwingflybase.oregonstate.edu/query>. ^b^ Protein names base on *Drosophila melanogaster*

**Supplementary Table 3 New genes annotation database**

| Annotated databases | New Gene Number |
| --- | --- |
| COG | 57 |
| GO | 208 |
| KEGG | 87 |
| Swiss-Prot | 163 |
| NR | 259 |
| All | 259 |

**Supplementary Table 4 The ovaries developmental status after 15 days of diapause and non-diapause inducing condition**

| **Sample sizes (n)** | | | | | | | | | |
| --- | --- | --- | --- | --- | --- | --- | --- | --- | --- |
|  | **A** | | | **B** | | | **C** | | |
|  | **Total number**  **of females** | **The ovaries developmental status rate (%)** | | **Total number**  **of females** | **The ovaries developmental status**  **rate (%)** | | **Total number**  **of females** | **The ovaries developmental status**  **rate (%)** | |
|  |  | **before stage 7**  **(previtellogenesis)** | **stage 7 or later (vitellogenesis)** |  | **before stage 7**  **(previtellogenesis)** | **stage 7 or later (vitellogenesis)** |  | **before stage 7**  **(previtellogenesis)** | **stage 7 or later (vitellogenesis)** |
| **8L:16D** | 35 | 94.29 | 5.71 | 38 | 97.37 | 2.63 | 36 | 94.45 | 5.55 |
| **16L:8D** | 36 | 11.11 | 88.89 | 37 | 10.81 | 89.19 | 39 | 7.69 | 92.31 |

The different letters (A, B, C) denote independent experiments.
